# Heterogeneous nuclear ribonucleoprotein A2/B1 promotes myocardial fibrosis by regulating the miR‐221‐3p/FOXO4‐mediated inflammation

**DOI:** 10.1002/ctm2.1616

**Published:** 2024-03-11

**Authors:** Xuping Li, Shuotao Shi, Zipei Li, Ying Wang, Xiaoxiao Qi, Rong Zhang, Zhongqiu Liu, Yuanyuan Cheng

**Affiliations:** ^1^ School of Pharmaceutical Sciences Joint Laboratory for Translational Cancer Research of Chinese Medicine of the Ministry of Education of the People's Republic of China, Guangdong Key Laboratory for translational Cancer research of Chinese Medicine, International Institute for Translational Chinese Medicine, School of Pharmaceutical Sciences, Guangzhou University of Chinese Medicine Guangzhou China

Dear Editor,

Myocardial fibrosis (MF) is the pathological basis of multiple cardiovascular diseases.^1^ Heterogeneous nuclear ribonucleoprotein A2/B1 (hnRNPA2B1), a member of the hnRNP family, participates in transcription and RNA metabolism by RNA‐binding. The process includes reading m6A‐marked messenger RNAs (mRNAs) and mediating microRNA (miRNA) maturation.^2^ Recently, hnRNPA2B1 was shown to be increased in the blood of acute myocardial infarction patients,^3^, ^4^ as well as upregulated in MF in an isoproterenol (ISO)‐induced model and ISO‐treated primary cardiac fibroblasts (Figure S1, S2C–F). In this study, we investigated a novel role for hnRNPA2B1 in MF.

In hnRNPA2B1 knockout (eKO) mice, the heart‐to‐body weight ratio was significantly reduced after ISO injection (Figure 1B). Echocardiography analysis showed a higher LV ejection fraction (%) from 45.93 ± 3.78 to 61.67 ± 1.51 and shortening fraction (FS%, 22.51 ± 2.08 to 32.03 ± 0.99) in the hnRNPA2B1 eKO mice than in the wild‐type (WT) mice after ISO stimulation (Figure 1C,D). HE staining and Masson staining results showed that the deletion of hnRNPA2B1 significantly reduced ISO‐induced inflammatory infiltration and collagen deposition (Figure 1E,F). Western blotting analysis also confirmed that the loss of hnRNPA2B1 significantly reduced the expression of cardiac fibrosis (CF) biomarkers, including collagen I/III, transforming growth factor beta 1 (TGF‐β1) and alpha‐smooth muscle actin (α‐SMA), induced by ISO (Figure 1G–L). The above experimental data suggest that the deletion of hnRNPA2B1 improves cardiac function and suppresses CF in ISO‐induced mice.

**FIGURE 1:**
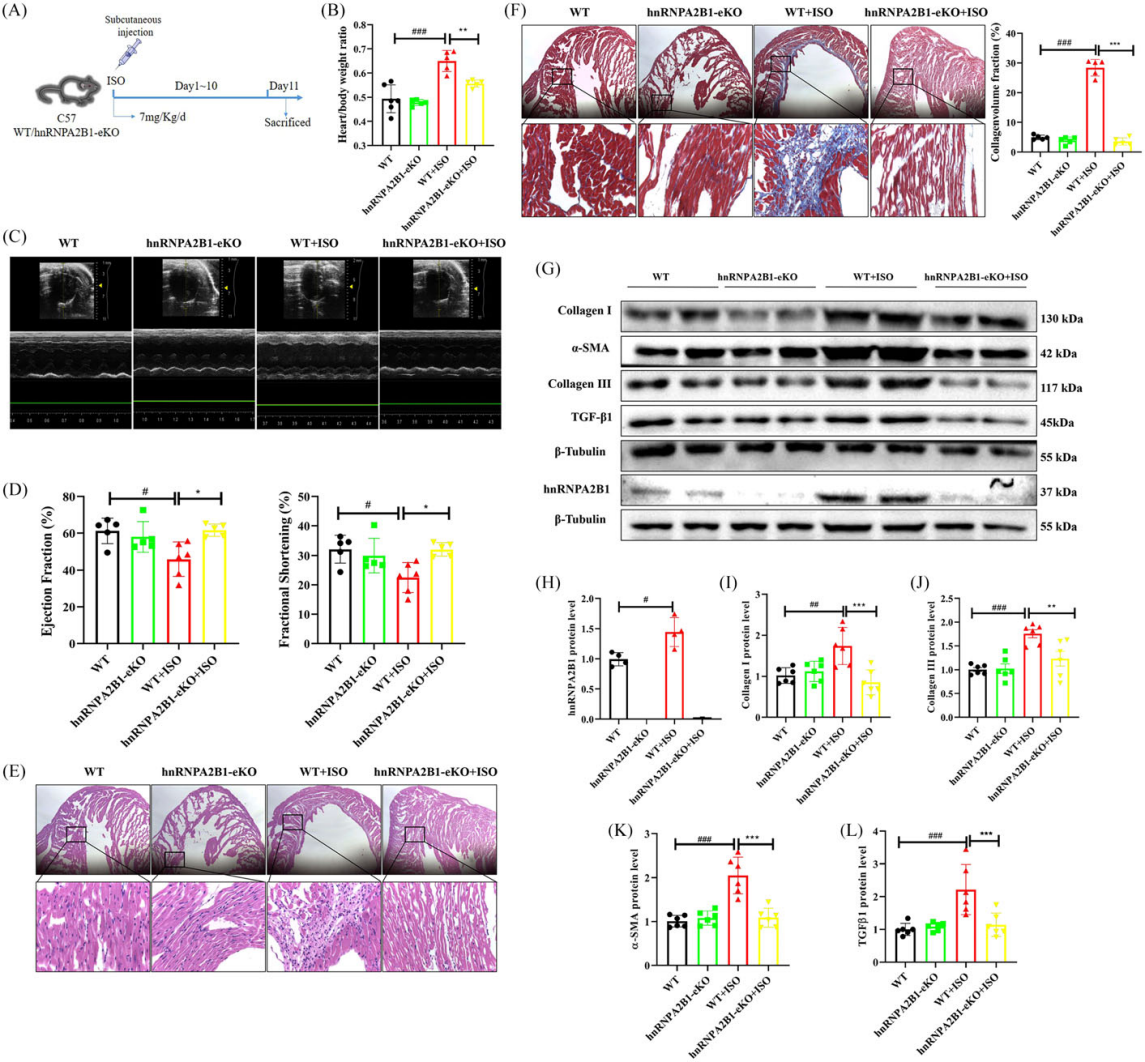
Heterogeneous nuclear ribonucleoprotein A2/B1 (hnRNPA2B1) knockout attenuated isoproterenol (ISO)‐induced myocardial fibrosis (MF) in vivo. (A) Scheme of the animal model. (B) hnRNPA2B1 knockout decreased the heart‐to‐body weight ratio. *n* = 5, ###*p *< .001, the wild‐type (WT) versus WT+ISO group;**p < .01, the hnRNPA2B1‐eKO+ISO versus WT+ISO group by one‐way ANOVA followed by Tukey's test. (C, D) hnRNPA2B1 knockout improved cardiac function after ISO stimulation. *n* = 5–6, #*p *< .05, the WT versus WT+ISO group;*p < .05, the hnRNPA2B1‐eKO+ISO versus WT+ISO group by one‐way ANOVA followed by Tukey's test. (E) H&E staining. Scale bars: 500 µm, 50 µm. (F) Masson staining, scale bar: 500 µm, 50 µm. The collagen volume fraction was analysed by Image J software. *n* = 5, ^###^ *p *< .001, the WT versus WT+ISO group; ***p < .001, the hnRNPA2B1‐eKO+ISO versus WT+ISO group by one‐way ANOVA followed by Tukey's test. (G–L) hnRNPA2B1 knockout reduced the expression of MF‐related markers. *n* = 6, ^#^ *p *< .05, ^##^ *p *< .01, ^###^ *p *< .001, the WT versus WT+ISO group;**p < .01, ***p < .001, the hnRNPA2B1‐eKO+ISO versus WT+ISO group by one‐way ANOVA followed by Tukey's test.

Cardiac fibroblasts’ proliferation and activation are associated with the development of MF.^5^ To study whether hnRNPA2B1 is involved in cardiac fibroblast proliferation and activation, we knocked down and overexpressed hnRNPA2B1 in primary cardiac fibroblasts by pretransfection of small interfering RNA (siRNA)‐hnRNPA2B1 and hnRNPA2B1 cDNA plasmids (Figure 2A and Figure S3A). The results of the EdU assay (Figure 2B and Figure S3B) showed that hnRNPA2B1 knockdown significantly decreased EdU+ cells after ISO stimulation, while hnRNPA2B1 overexpression increased EdU+ cells. The MTT assay also showed that the growth curve of the siRNA‐hnRNPA2B1+ISO group was decreased, however, hnRNPA2B1 overexpression promoted the growth of cardiac fibroblasts after ISO stimulation (Figure 2C and Figure S3C), which indicate that hnRNPA2B1 enhances the proliferation of cardiac fibroblasts. After cardiac fibroblast activation, extracellular matrix proteins such as α‐SMA and collagen I/III are highly expressed, thus, we used western blotting to detect their expression after hnRNPA2B1 knockdown or overexpression. HnRNPA2B1 silencing markedly decreased the expression of collagen I/III, TGF‐β1 and α‐SMA, while hnRNPA2B1 overexpression dramatically increased the expression of these MF‐related proteins (Figure 2D–H and Figure S3D–H). These data suggested that hnRNPA2B1 promotes the ISO‐stimulated tendency towards cardiac fibroblast fibrosis.

**FIGURE 2:**
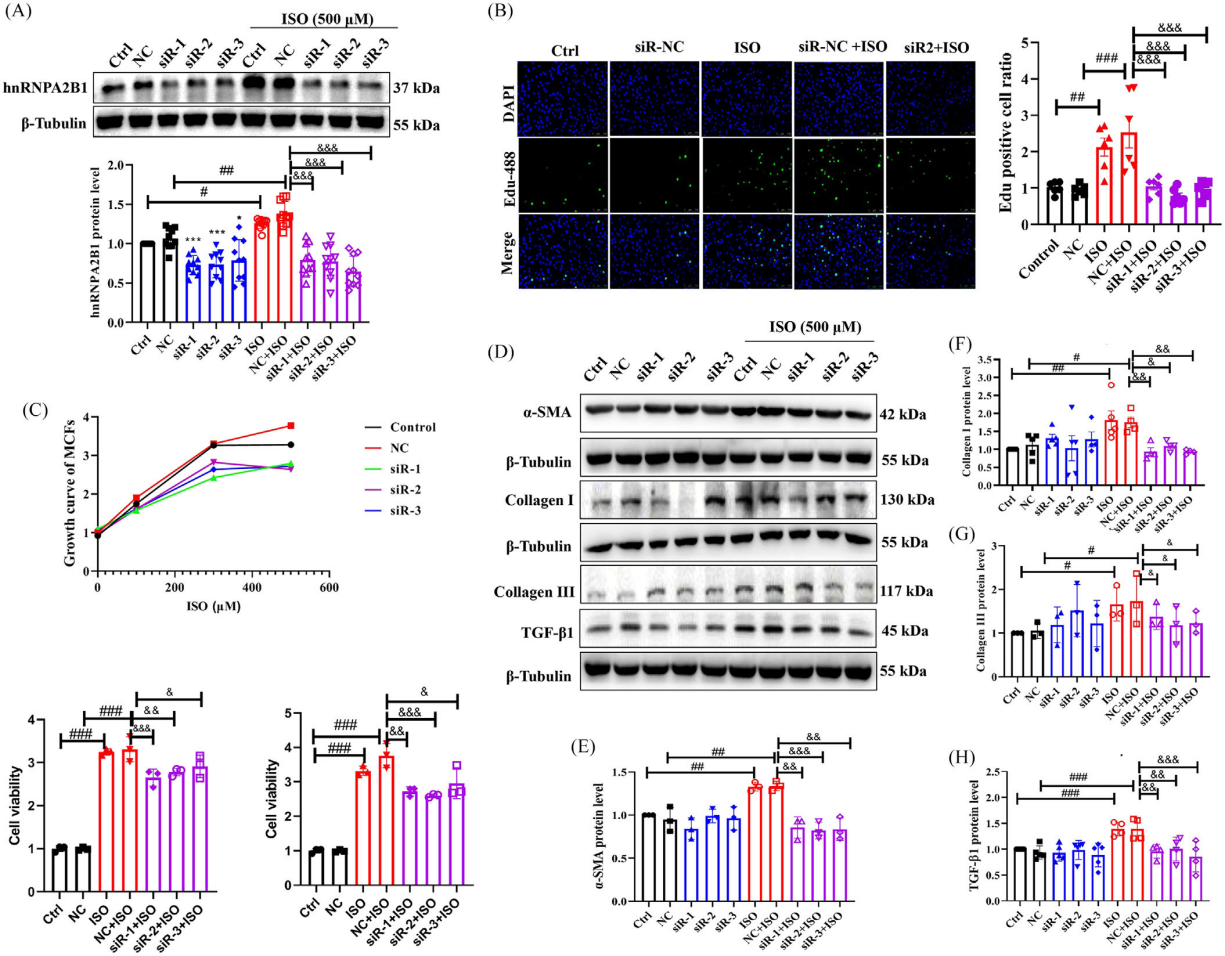
Heterogeneous nuclear ribonucleoprotein A2/B1 (hnRNPA2B1) knockdown inhibited isoproterenol (ISO)‐induced myofibroblast proliferation and activation. (A) The expression of hnRNPA2B1 protein was measured using WB analysis after transfection of hnRNPA2B1 siRNA. *n* = 10, *p < .05, ***p < .001; #*p *< .05, ^##^ *p *< .01; ^&&&^ *p *< .001 by one‐way ANOVA followed by Tukey's test. (B) The effect of hnRNPA2B1 knockdown on the proliferation of myofibroblasts detected by the EdU incorporation assay. Scale bar: 100 µm. *n* = 6, ^##^ *p *< .01, ^###^ *p *< .001; ^&&&^ *p *< .001 by one‐way ANOVA followed by Tukey's test. (C) The effect of hnRNPA2B1 knockdown on the growth curve of myofibroblasts was measured by the MTT assay. *n* = 3, ^###^ *p *< .001; ^&^ *p *< .05, ^&&^ *p *< .01, ^&&&^ *p *< .001 by one‐way ANOVA followed by Tukey's test. (D–H) The effect of hnRNPA2B1 knockdown on the expression of cardiac fibrosis‐related markers (ɑ‐SMA, Collagen I/III and TGFβ1). Data are the mean ± SD, *n* = 3–5. ^#^ *p *< .05, ^##^ *p *< .01, ^###^ *p *< .001; ^&^ *p *< .05, ^&&^ *p *< .01, ^&&&^ *p *< .001 by one‐way ANOVA followed by Tukey's test.

Considering that hnRNPA2B1 can read m6A‐marked miRNA and mediate miRNA maturation, we collected data on miRNAs regulated by hnRNPA2B1^2^ and miRNAs that were involved in CF (Table S1) from the reported literature. MiR‐126, miR‐181a, miR‐210, miR‐99b and miR‐221 were screened out (Figure 3A) and determined by quantitative real‐time polymerase chain reaction (qRT‐PCR), however, only three of them were detected. miR‐99b‐3p, miR‐221‐3p and miR‐210‐5p expression were upregulated in the ISO‐induced left ventricle of mice, and miR‐221‐3p was more significant (Figure 3B). Importantly, in hnRNPA2B1 eKO mice, the elevated miR‐99b‐3p, miR‐210‐5p and miRNA‐221‐3p induced by ISO were blocked compared with that in WT mice (Figure 3C). Moreover, the miR‐99b‐3p, miR‐210‐5p and miR‐221‐3p levels in ISO‐induced primary cardiac fibroblasts were significantly decreased after hnRNPA2B1 silencing (Figure 3D). Considering the significant changes in miRNAs at the animal and cell levels regulated by hnRNPA2B1, we selected miR‐221‐3p for mechanistic discussion.

**FIGURE 3:**
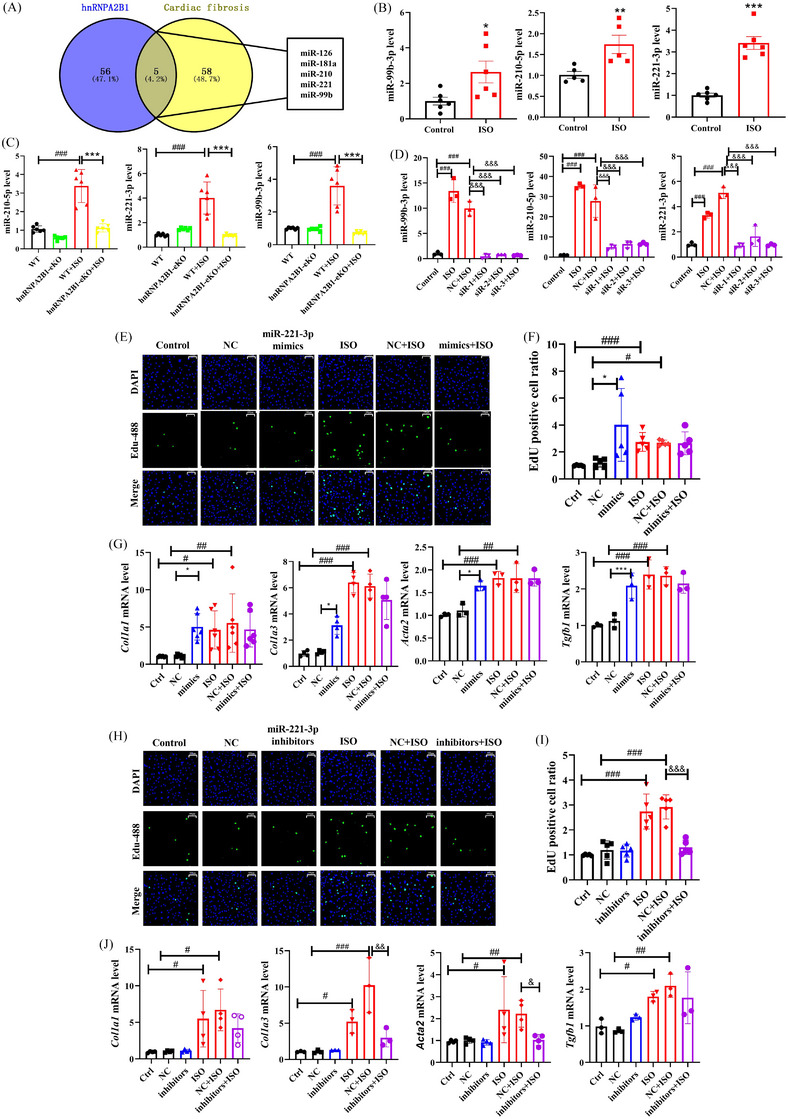
miR‐221‐3p is a key regulator of heterogeneous nuclear ribonucleoprotein A2/B1 (hnRNPA2B1) in myocardial fibrosis (MF). (A) Venn diagram. (B) The expression of miR‐99‐3p, miR‐210‐5p and miR‐221‐3p in the heart tissue of isoproterenol (ISO)‐induced MF mice. *n* = 5–6, *p < .05, **p < .01, ***p < .001, the control versus ISO group. (C) hnRNPA2B1 knockout decreased the expression of miR‐99‐3p, miR‐210‐5p and miR‐221‐3p in vivo. *n* = 6, ^###^ *p *< .001, the wild‐type (WT) versus WT+ISO group; ***p < .001, the hnRNPA2B1‐eKO+ISO versus WT+ISO group. (D) hnRNPA2B1 silencing reduced the expression of miR‐99‐3p, miR‐210‐5p and miR‐221‐3p in ISO‐induced myofibroblasts. *n* = 3, ^###^ *p *< .001, the control versus ISO or NC+ISO group; ^&&&^ *p *< .001, the hnRNPA2B1‐siR+ISO versus NC+ISO group. (E, F) miR‐221‐3p mimics promoted ISO‐induced myofibroblast proliferation. An EdU assay was used to determine the proliferation of myofibroblasts. *n* = 5, *p < 0.05, the mimics versus the NC group; ^#^ *p *< .05, ^###^ *p *< .001, the Ctrl or NC group versus the ISO or NC+ISO group by one‐way ANOVA followed by Dunnett's test. (G) miR‐221‐3p mimics promoted myofibroblast activation. *n* = 3–6, *p < .05, ***p < .001, the mimics versus the NC group; ^#^ *p *< .05, ^##^ *p *< .01, ^###^ *p *< .001, the Ctrl or NC group versus the ISO or NC+ISO group by one‐way ANOVA followed by Dunnett's test. (H and I) miR‐221‐3p inhibitor suppressed ISO‐induced myofibroblast proliferation. An EdU assay was used to determine the proliferation of myofibroblasts. *n* = 5, ^###^ *p *< .001, the Ctrl or NC group versus the ISO or NC+ISO group; ^&&&^ *p *< .001, the NC+ISO versus the inhibitor+ISO group by one‐way ANOVA followed by Dunnett's test. (J) miR‐221‐3p inhibitors suppressed ISO‐induced myofibroblast activation. *n* = 3–4, ^#^ *p *< .05, ^##^ *p *< .01, ^###^ *p *< .001, the Ctrl or NC group versus the ISO or NC+ISO group; ^&^ *p *< .05, ^&&^ *p *< .01, the NC+ISO versus the inhibitor+ISO group by one‐way ANOVA followed by Dunnett's test.

To evaluate the function of miR‐221‐3p in the activation of CF, transfection of miR‐221‐3p mimics and inhibitors into primary cardiac fibroblasts was performed. Treatment with miRNA‐221‐3p mimics enhanced the proliferation of cardiac fibroblasts and increased the expression of CF‐related genes (*Col1a1*, *Col3a1, Tgfb1* and *Acta2*, Figure 3E–G), while miRNA‐221‐3p inhibitors retrained the proliferation and those genes in ISO‐induced primary cardiac fibroblasts (Figure 3H–J).

*Foxo4*, one of the widely expressed forkhead box (Fox) transcription factor O family members, regulates oxidative stress/immune response, metabolism and apoptosis.^6^ We detected *Foxo4* mRNA levels, which are highly expressed in the ISO‐induced heart (Figure 4A). Subsequently, two binding sites of miR‐221‐3p were predicted in the 3′‐UTR of Foxo4 using StarBase (Figure 4B). Thus, we speculated that *Foxo4* could be a target of miR‐221‐3p involved in CF. A luciferase reporter assay exhibited that miR‐221‐3p represses *Foxo4* by binding its 3′‐UTR. Mutation of the two binding sites of miR‐221‐3p eliminated the repression (Figure 4C), confirming the direct targeting of Foxo4 by miR‐221‐3p. Furthermore, qPCR analysis confirmed that treatment with miR‐221‐3p mimics suppressed *Foxo4* mRNA expression in primary cardiac fibroblasts (Figure 4D), while miR‐221‐3p inhibitors increased *Foxo4* expression (Figure 4E). In addition, *Foxo4* mRNA expression was significantly elevated in hnRNPA2B1 eKO mice after ISO stimulation (Figure 4F). Consistent with the in vivo results, *Foxo4* expression was increased after hnRNPA2B1 silencing in primary cardiac fibroblasts (Figure 4G).

**FIGURE 4:**
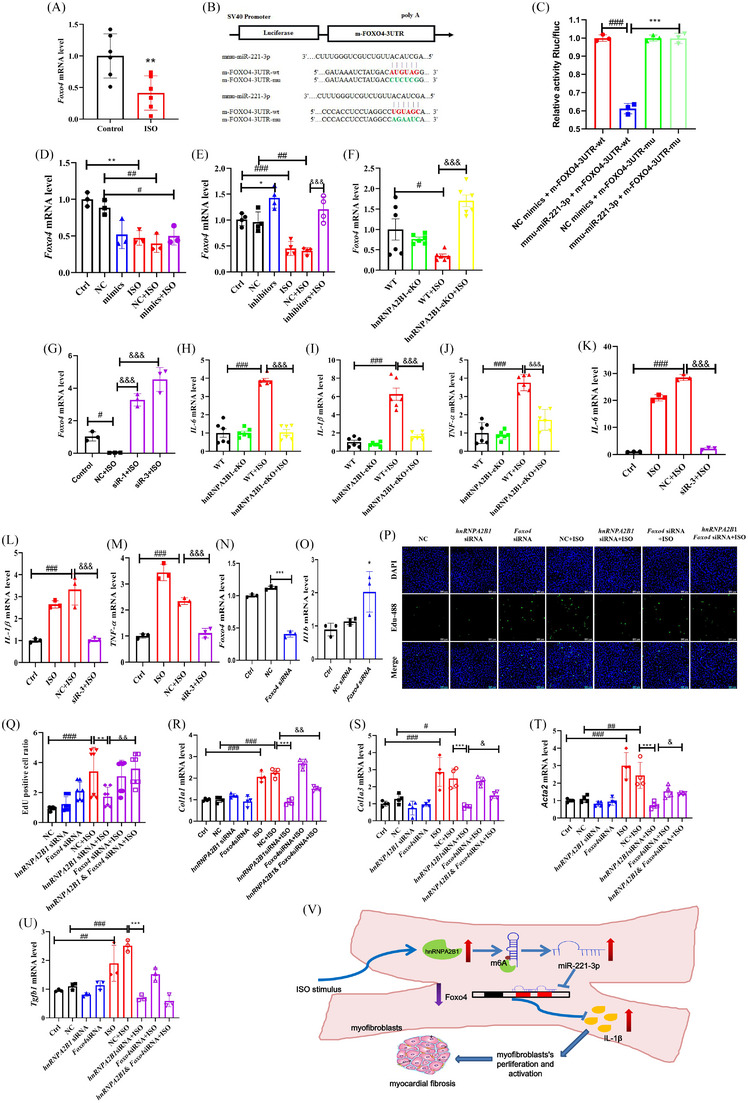
*Foxo4* is the target of miR‐221‐3p. (A) The *Foxo4* mRNA level in the isoproterenol (ISO)‐induced myocardial fibrosis (MF) mouse model. *n* = 6, **p < .01, the control versus ISO group by unpaired Student's t‐test. (B) Schematic representation of the wild‐type and mutant‐type binding site between the 3′UTR of Foxo4 and miR‐221‐3p. (C) Relative luciferase activity of 3′UTR‐Foxo4‐luc constructs in HEK293T cells after transfection of miR‐221‐3p mimics/NC. *n* = 3, ***p < .001, miR‐221‐3p mimics + Foxo‐3UTR‐wt versus NC mimics + Foxo‐3UTR‐wt. (D and E) The expression of *Foxo4* mRNA in ISO‐induced primary myofibroblasts when using miR‐221‐3p mimics or inhibitors. *n* = 3–4, *p < .05, miR‐221‐3p mimics or inhibitors versus NC, ^##^ *p *< .01, ^###^ *p *< .001, the control or NC versus ISO or NC+ISO group; ^&&&^ *p *< .001, NC+ISO versus miR‐221‐3p inhibitors + ISO by one‐way ANOVA followed by Dunnett's test. (F) The expression of *Foxo4* mRNA in the heart tissue of ISO‐induced hnRNPA2B1 knockout mice. *n* = 6, #*p *< .05, the WT versus WT+ISO group; *^&&&^p *< .001, the hnRNPA2B1‐eKO+ISO versus WT+ISO group by one‐way ANOVA followed by Dunnett's test. (G) The expression of *Foxo4* mRNA in ISO‐induced primary myofibroblasts after hnRNPA2B1 knockdown*. n* = 3, #*p *< .05, the control versus ISO or NC+ISO group; ^&&&^ *p *< .001, the hnRNPA2B1‐siR+ISO versus NC+ISO group. (H–J) The level of inflammatory mediators (*Il6, Tnfa* and *Il1b*) at mRNA level in the heart tissue of ISO‐induced hnRNPA2B1 knockout mice. *n* = 6, ^###^ *p *< .001, the WT versus WT+ISO group; *^&&&^p *< .001, the hnRNPA2B1‐eKO+ISO versus WT+ISO group by one‐way ANOVA followed by Dunnett's test. (K–M) The level of inflammatory mediators (*Il6, Tnfa* and *Il1b*) at mRNA level in ISO‐induced primary myofibroblasts after hnRNPA2B1 knockdown. *n* = 3, ^###^ *p *< .001, the control versus ISO or NC+ISO group; ^&&&^ *p *< .001, the hnRNPA2B1‐siR+ISO versus NC+ISO group by one‐way ANOVA followed by Dunnett's test. (N) The level of *Foxo4* mRNA level after *Foxo4* siRNA transfection. (O) The level of *Il1b* mRNA level in primary myofibroblasts after *Foxo4* knockdown. *n* = 3, *p < .05, the NC siRNA versus the *Foxo4* siRNA group by one‐way ANOVA followed by Dunnett's test. (P, Q) *Foxo4* silencing blocked the negative effect of hnRNPA2B1 knockdown on the proliferation of myofibroblasts. An EdU assay was used to determine the proliferation of myofibroblasts. *n* = 7, ^##^ *^#^p *< .001, the NC group versus The NC+ISO group; **p < .01, the NC+ISO group versus the *hnRNPA2B1* siRNA+ISO group; ^&&^ *p *< .01, the *hnRNPA2B1* siRNA+ISO group versus the *hnRNPA2B1* siRNA + *Foxo4* siRNA+ ISO group by one‐way ANOVA followed by Dunnett's test. (R–U) *Foxo4* silencing blocked the negative effect of hnRNPA2B1 knockdown on the activation of myofibroblasts. *n* = 3–4, ^##^ *p *< .05, ^#^ *^#^p *< .01, ^##^ *^#^p *< .001, the NC group versus The NC+ISO group; ***p < .001, the NC+ISO group versus the *hnRNPA2B1* siRNA+ISO group; ^&^ *p *< .05, ^&&^ *p *< .01, the *hnRNPA2B1* siRNA+ISO group versus the *hnRNPA2B1* siRNA + *Foxo4* siRNA+ ISO group by one‐way ANOVA followed by Tukey's test. (V) The potential mechanism of hnRNPA2B1 on cardiac fibrosis.

Foxo4 is related to the inflammatory response.^7^, ^8^ Thus, we evaluated the levels of proinflammatory mediators (*Il6, Il1b* and *Tnfa*). Their mRNA levels were increased in the ISO‐induced left ventricles of mice and primary cardiac fibroblasts (Figure S4A–C). In addition, the *Il6, Il1b* and *Tnfa* mRNA levels markedly declined in hnRNPA2B1 eKO mice under ISO stimulation (Figure 4H–J). Consistent with the in vivo results, *Il6, Il1b* and *Tnfa* mRNA levels were also reduced after hnRNPA2B1 silencing in primary cardiac fibroblasts (Figure 4K–M). Interestingly, Foxo4 silencing significantly increased the mRNA level of *Il1b* but did not affect *Il6* and *Tnfa* (Figure 4N–O and Figure S4D,E).

To confirm the role of Foxo4 in hnRNPA2B1‐mediated MF, we performed a rescue assay. in hnRNPA2B1‐silenced myofibroblasts, Foxo4 knockdown increased EdU+ cells numberAs (Figure 4P,Q). In addition, Foxo4 knockdown blocked the negative regulation of hnRNPA2B1 silencing on MF‐related gene expression (*Col1a1, Col3a1, Acta2* and *Tgf1b*, Figure 4R–U). The above results indicated that Foxo4 is a key regulator in hnRNPA2B1‐mediated CF.

In conclusion, our study indicated that hnRNPA2B1 participated in ISO‐induced MF, and the mechanism was associated with regulating the miR‐221‐3p/Foxo4‐mediated inflammatory response and myofibroblast activation (Figure 4V).

## AUTHOR CONTRIBUTIONS

Xuping Li and Shuotao Shi performed the experiments, analyzed the data and wrote the manuscript; Zipei Li, Ying Wang and Xiaoxiao Qi assisted in animal experiments; Rong Zhang provided technical support and review the manuscript; Zhongqiu Liu and Yuanyuan Cheng supervised the project, and edited the manuscript. All authors read and approved the final manuscript.

## CONFLICT OF INTEREST STATEMENT

The authors declare no conflict of interest.

## FUNDING INFORMATION

We would like to thank the Guangdong Key Laboratory for Translational Cancer Research of Chinese Medicine for providing the platform. This work was supported by the National Natural Science Foundation of China (Nos. 82074053, 81930114, 82374070 and U22A20368), the Natural Science Foundation of Guangdong Province (No. 2019A1515010211), Key‐Area Research and Development Program of Guangdong Province (No. 2020B1111100004) and Guangzhou Association for Science and Technology Youth Talent Support Program (No. QT‐2023‐011).

## ETHICS STATEMENT

The animal expriments were approved by the Guangzhou University of Chinese Medicine Animal Care and Use Committee and the approved number is 20221035 and IITCM‐20190149.

## Supporting information

Supporting Information

Supporting Information

## References

[1] Gyongyosi M , Winkler J , Romas I , et al. Myocardial fibrosis: biomedical research from bench to bedside. Eur J Heart Fail. 2017;19:177‐191. 28157267 10.1002/ejhf.696 PMC5299507

[2] Alarcon CR , Goodarzi H , Lee H , et al. HNRNPA2B1 is a mediator of m(6)A‐dependent nuclear RNA processing events. Cell. 2015;162:1299‐1308. 26321680 10.1016/j.cell.2015.08.011 PMC4673968

[3] Wang X , Wu Y , Guo RY , et al. Comprehensive analysis of N6‐methyladenosine RNA methylation regulators in the diagnosis and subtype classification of acute myocardial infarction. J Immunol Res. 2022;2022:5173761. 36061306 10.1155/2022/5173761 PMC9433256

[4] Liu Z , Wang L , Xing QC , et al. Identification of GLS as a cuproptosis‐related diagnosis gene in acute myocardial infarction. Front Cardiovasc Med. 2022;9:1016081. 36440046 10.3389/fcvm.2022.1016081 PMC9691691

[5] Frangogiannis NG , fibrosis Cardiac . Cardiac fibrosis. Cardiovasc Res. 2021;117:1450‐1488. 33135058 10.1093/cvr/cvaa324 PMC8152700

[6] Zhu M , Goetsch SC , Wang ZN , et al. FoxO4 promotes early inflammatory response upon myocardial infarction via endothelial Arg1. Circ Res. 2015;117:967‐977. 26438688 10.1161/CIRCRESAHA.115.306919 PMC4710860

[7] Huang HB , Dong JF , Jiang JQ , et al. The role of FOXO4/NFAT2 signaling pathway in dysfunction of human coronary endothelial cells and inflammatory infiltration of vasculitis in Kawasaki disease. Front Immunol. 2022;13:1090056. 36700213 10.3389/fimmu.2022.1090056 PMC9869249

[8] Zhou W , Cao Q , Peng Y , et al. FoxO4 inhibits NF‐kappaB and protects mice against colonic injury and inflammation. Gastroenterology. 2009;137:1403‐1414. 19560465 10.1053/j.gastro.2009.06.049 PMC2764529

