# Supplementary material for: Heterogeneous nuclear ribonucleoprotein A2/B1 promotes myocardial fibrosis by regulating the miR‐221‐3p/FOXO4‐mediated inflammation
Source: Clin Transl Med. 2024 Mar 11;14(3):e1616. doi: 10.1002/ctm2.1616 (PMC10928344; doi:10.1002/ctm2.1616)
Supplement: Supplementary file 1 — Supporting Information [file CTM2-14-e1616-s002.docx]

**HnRNPA2B1** **promotes** **myocardial fibrosis and proliferation of cardiac fibroblasts via the miR-221-3p/FOXO4-mediated inflammatory response** **in isoproterenol-induced mice**

Xuping Li^a,#^, Shuotao Shi^a,#^, Zipei Li^a^, Ying Wang^a^, Xiaoxiao Qi^a^, Rong Zhang^a^, Zhongqiu Liu^a,^*, Yuanyuan Cheng^a,*^

^a^Joint Laboratory for Translational Cancer Research of Chinese Medicine of the Ministry of Education of the People's Republic of China, Guangdong Key Laboratory for translational Cancer research of Chinese Medicine, International Institute for Translational Chinese Medicine, School of Pharmaceutical Sciences, Guangzhou University of Chinese Medicine, Guangzhou, Guangdong,

#Xuping Li and Shuotao Shi were equally contributed to the work.


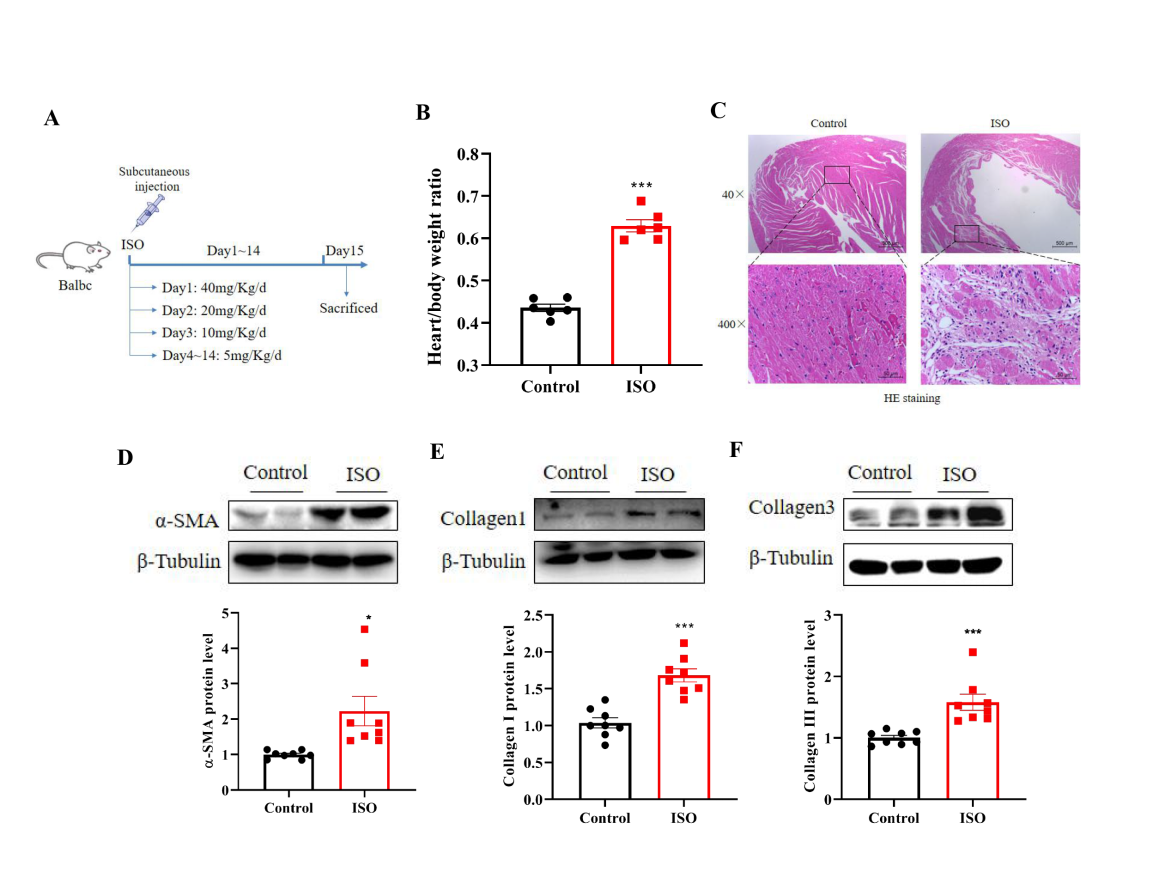


**Figure S1. The established myocardial fibrosis model induced by isoproterenol in BALBc mice.** (A) The flowchart for animal model. （B）The ratio of heart weight and body weight in ISO-induced mice model. n = 6, ****p*<0.001, the control vs. ISO group by unpaired Student’s t test.(C) The H&E staining. (D-F) The cardiac fibrosis related proteins expression in ISO-induced mice model. n = 8, **p*<0.05,****p*<0.001, the control vs. ISO group by unpaired Student’s t test.

**
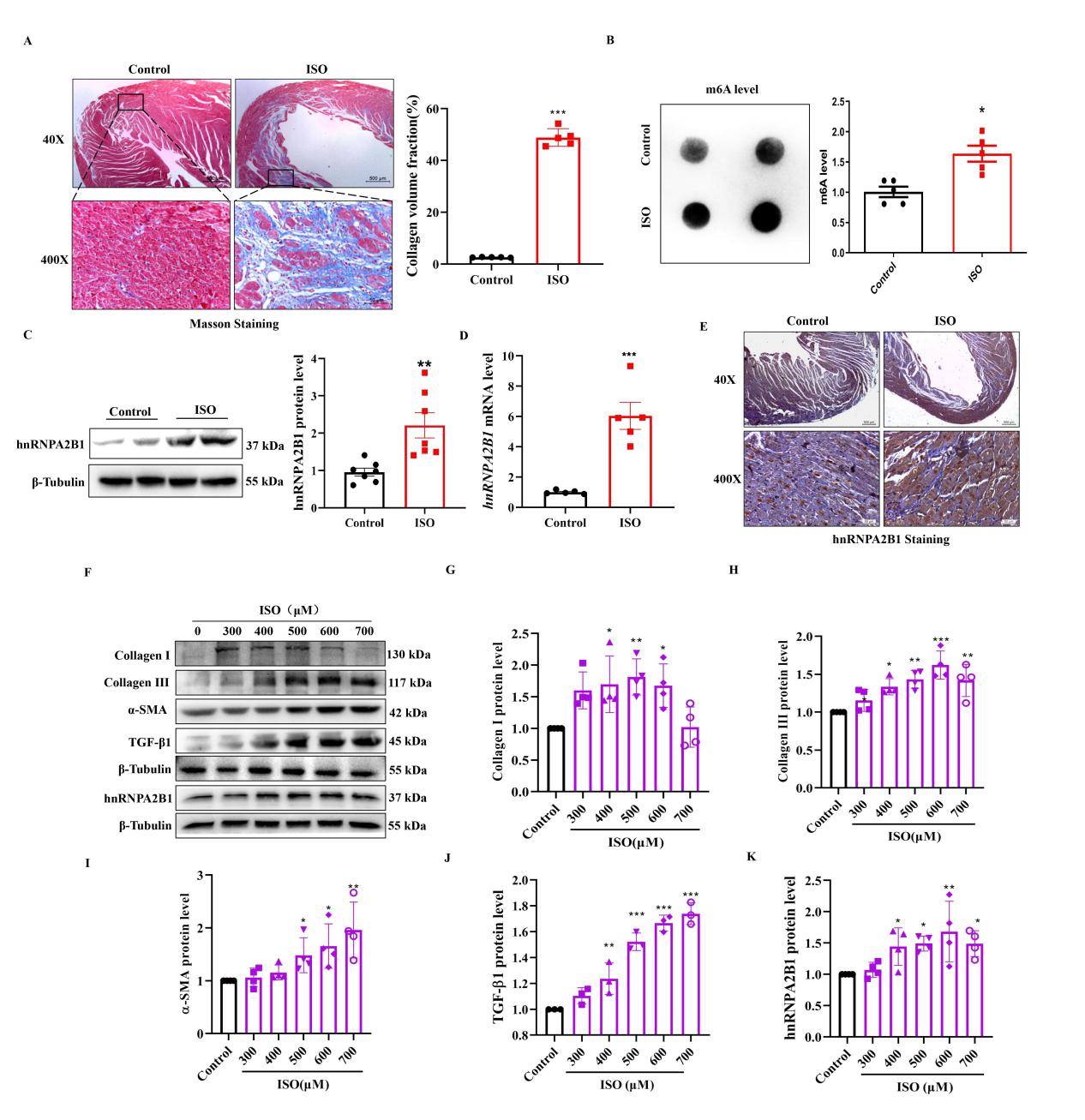
**

**Figure S2. hnRNPA2B1 was upregulated in the ISO-induced myocardial fibrosis model *in vivo* and *in vitro*.** (A) Established ISO-induced myocardial fibrosis mouse model. Masson staining, scale bar: 500 μm, 50 μm. The collagen volume fraction was analysed. n = 6, ****p*<0.001, the control vs. ISO group by unpaired Student’s t test. (B) The m6A level in the ISO-induced MF mouse model. n = 4, **p*<0.05, the control vs. ISO group by unpaired Student’s t test. (C) The expression of hnRNPA2B1 in the ISO-induced MF mouse model. n = 5, **p*<0.05, the control vs. ISO group by unpaired Student’s t test. (D) The mRNA level of *hnrnpa2b1* in the ISO-induced MF mouse model. n = 5, ****p*<0.001, the control vs. ISO group by unpaired Student’s t test. (E) Immunohistochemical staining of hnRNPA2B1 in heart tissue. Scale bars: 500 μm, 50 μm. (F-K) The expression of MF-related markers and hnRNPA2B1 in ISO-induced primary myofibroblasts. n = 3~4, **p*<0.05, **p*<0.01,**p*<0.001, the control vs. different concentrations of ISO groups by one-way ANOVA followed by Dunnett’s test.


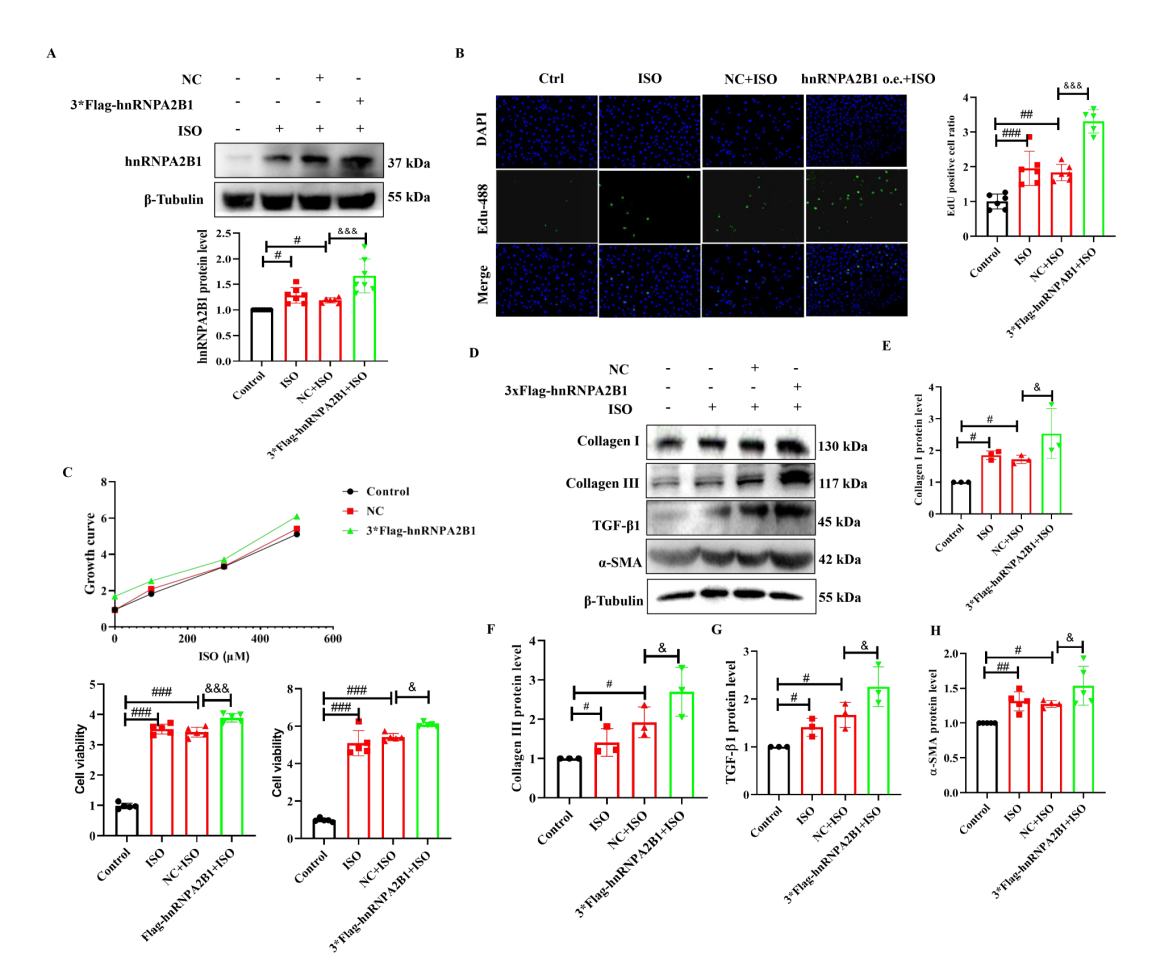


**Figure S3. hnRNPA2B1 overexpression promoted ISO-induced myofibroblast proliferation and activation.** (A) The expression of hnRNPA2B1 protein was measured using WB analysis after transfection of hnRNPA2B1-overexpressing vectors. n = 7. **p*<0.05; ^&&&^*p*<0.001 by one-way ANOVA followed by Tukey’s test. (B) The effect of hnRNPA2B1 overexpression on the proliferation of myofibroblasts detected by the EdU incorporation assay. Scale bar: 100 μm. n = 6, ##*p*<0.01, ###*p*<0.001; ^&&&^*p*<0.001 by one-way ANOVA followed by Tukey’s test. (C) The effect of hnRNPA2B1 overexpression on the growth curve of myofibroblasts measured by the MTT assay. n = 5, ###*p*<0.001; ^&^*p*<0.05, ^&&^*p*<0.01, ^&&&^*p*<0.001 by one-way ANOVA followed by Tukey’s test. (D-H) The effect of hnRNPA2B1 knockdown on the expression of cardiac fibrosis-related markers (ɑ-SMA, Collagen I/III, TGFβ1). Data are the mean ± SD, n = 3~5. #*p*<0.05, ##*p*<0.01; ^&^*p*<0.05 by one-way ANOVA followed by Tukey’s test.


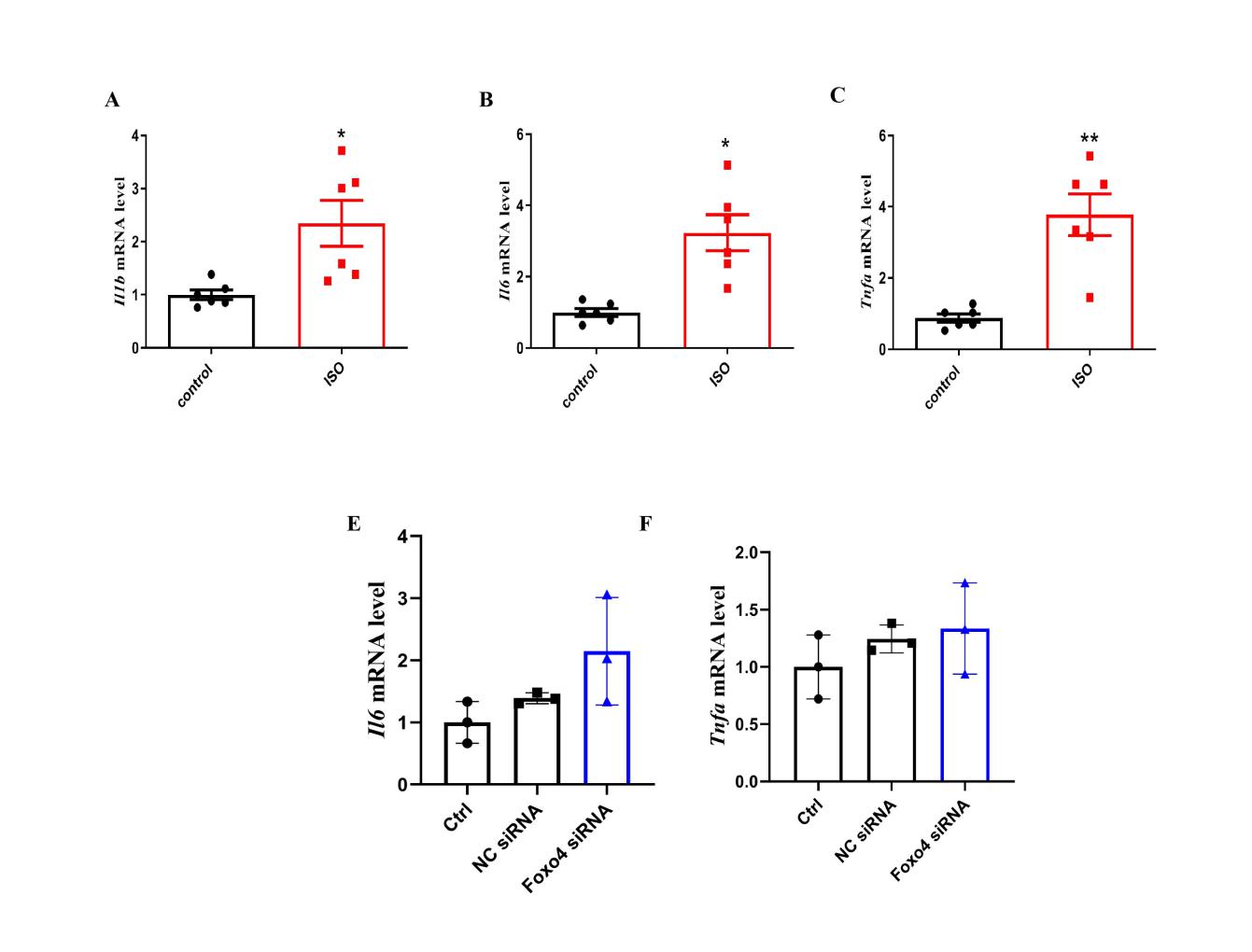


**Figure S4 The expression of inflammatory mediators in the ISO-induced MF mouse model.** (A-C) The level of inflammatory mediators (*Il6, Tnfa, Il1b*) at mRNA level in the ISO-induced MF mouse model. n = 6,**p*<0.05, ***p*<0.01, the control vs. ISO group by unpaired Student’s t test. (D-E) The level of *Il6 and Tnfa* mRNA level in primary myofibroblasts afte*r Foxo4* knockdown. n = 3, **p*<0.05, the NC siRNA vs. the *Foxo4* siRNA group by one-way ANOVA followed by Dunnett’s test.

**Methods and Materials**

**Reagents and antibodies**

The sources of antibodies used in western blotting analysis were as follows: anti-α-SMA (19245S) was purchased from Cell Signaling Technology (CA, USA); anti-collagen I (bs-10423R), anti-collagen III (bs0549R) and anti-β-tubulin (bs-0210R) were purchased from Bioss Technology (Beijing, China); and anti-hnRNPA2B1 (14813-1-AP) was purchased from Proteintech (IL, USA). The miRNA cDNA first-strand synthesis reagent (KR211) and miRcute enhanced miRNA fluorescence quantitative kit were purchased from TIANGEN (Beijing, China); the Evo M-MLV cDNA reverse transcription kit (AG11728) and SYBR Green Pro Taq HS Fluorescent Quantitative Detection Kit (AG11718) were purchased from AGbio (Hunan, China). The SP-(mouse/rabbit IGg)-POD kit (SP0041) used in chemical experiments was obtained from Solarbio (Beijing, China). The BeyoClick EdU-488 Cell Proliferation Detection Kit (C0071S) was purchased from Beyotime Biotechnology (Shanghai, China). All synthetic primers were purchased from Sangon Biotech (Shanghai, China), and the primer sequences were as follows: *hnRNPA2B1* (forward: TTCCAGACTGCCTATCGGTAA, reverse: CAGGGTAGTTGAGCCAAAACG); *Foxo4* (forward: AGCAGGAGGTGGTGGTGTATCAG, reverse: GATGGGTCTTTGTCAGCAGGAGAAG); *Il6* (forward: GGTATCCTCTGTGAAGTCTCCTCTCC, reverse: AGTTGCCTTCTTGGGACTGATGTTG); *Il1β* (forward: TCCACGGGCAAGACATAGGTAGC, reverse: AATCTCACAGCAGCATCTCGACAAG); *Tnfa* (forward: CCTCCGCTTGGTGGTTTGCTAC, reverse: ATGGGCTCCCTCTCATCAGTTCC); *Tgfb1* (forward: CTGGCACTGCTTCCCGAATGTC, reverse: GACCGCAACAACGCAATCTATGAC).

**HnRNPA2B1 eKO mouse gene identification protocol**

KO mouse identification primer sequences are as follows. The forward primer: TCGTTGTGCAGTCATAGGTCC; the reverse primer: TTCGGATTCATCCTTGGGCT. Mix the prepared gene (2 μL) samples with 2×Taq Plus Master Mix(10μL), ddH2O (7μL), forward primer (0.5μL) and reverse primer (0.5μL). The PCR program is as follows: 1.94℃ for 3mins; 2. 98℃ for 15 secs, 60℃ for 15secs, 68℃ for 1min; repeat step 2 for 35 cycles; 3. 68 for 15mins. Prepared PCR samples were identified by electrophoresis using 1.0% agarose gel.

**Animal**

HnRNPA2B1-eKO mice (C57BL/6 background) were generated by Shanghai Model Organisms Center, Inc., and identified by PCR (*Figure S2*). All procedures of animal experiments were approved by the Guangzhou University of Chinese Medicine Animal Care and Use Committee and followed institutional guidelines (permission number 20221035, approved date: 2022.10.25). Sixteen hnRNPA2B1-WT and hnRNPA2B1-eKO male mice aged 8 weeks were used for this study. In addition, BALBc mice aged 8 weeks and the newborn rats from the Laboratory Animal Center of Southern Medical University (Guangdong, China) were also used in the study (permission number IITCM-20190149, approved date: 2019.8.28). All animal experiments conform to the guidelines from Directive 2010/63/EU of the European Parliament on the protection of animals used for scientific purposes.

**ISO-induced myocardial fibrosis mouse model**

After 7 days of adaptive feeding, male BALBc mice (weight: 22-25 g) were subcutaneously injected with ISO dissolved in normal saline at the following doses: Day 1 (40 mg/kg), Day 2 (20 mg/kg/day), Day 3 (10 mg/kg/day), and Days 4-14 (5 mg/kg). Male C57BL/6 mice (hnRNPA2B1-eKO or hnRNPA2B1-WT mice, weight: 22-25 g) were adaptively fed for 7 days and administered ISO at a dose of 7 mg/kg/day by subcutaneous injection for 10 consecutive days. At the end of the experiment, all mice were anesthetized by 1.5% isoflurane and subjected to a VEVO 770 high-resolution imaging system (Visual Sonics Inc., Toronto, Canada) for echocardiographic assessment.

**Masson staining and H&E staining**

Cardiac tissues were fixed in 4% paraformaldehyde (PFA) for 24 hours, dehydrated by an alcohol gradient, embedded in paraffin and sectioned after rehydration. For Masson staining, the slices were stained with haematoxylin, ponceau acid stain and aniline blue solution. Then, the slices were mounted with neutral resin after dehydration. Finally, the nuclei were blue‒purple, the cytoplasm was pink, and the collagen fibres were blue. For H&E staining, the slices were stained with haematoxylin and eosin. Then, the nuclei are blue‒purple, and the cytoplasm and extracellular matrix are red.

**Immunohistochemistry**

Paraffin cardiac tissue sections were dewaxed, placed in 0.01 M citrate buffer solution at pH 6.0, and placed in a microwave oven for antigen heat retrieval. Then, the slices were immersed in PBS containing 3% H2O2 to decrease the influence of endogenous peroxidase. After that, the slices were blocked in PBST solution with 5% goat serum at RT for 1 hour and probed with hnRNPA2B1 antibody at 4 °C overnight. After washing, the slices were incubated with the enzyme-conjugated secondary antibody at RT for 1 hour. for 1 h. Finally, the chromogenic substrate DAB solution was dropped to colour, and the nuclei were counterstained with haematoxylin.

**Western blotting analysis**

After measurement of concentration, the protein samples were loaded on the polyacrylamide gel for electrophoresis and transferred to PVDF membranes. The PVDF membrane was blocked with 5% skim milk for 1 h and then incubated with primary antibodies (collagen I/III, ɑ-SMA, TGFβ1, hnRNPA2B1) overnight at 4 °C. After incubation with horseradish peroxidase (HRP)-labelled secondary antibodies for 1 h, enhanced chemiluminescence (ECL) reagent was used to detect the bands.

**Real-time fluorescent quantitative PCR (RT‒qPCR)**

The extracted RNA was reverse-transcribed by using miRNA cDNA first-strand synthesis reagent (KR211) and an Evo M-MLV cDNA reverse transcription kit (AG11728). The expression of miRNA and mRNA in the samples was detected by a miRcute enhanced miRNA fluorescence quantitative kit or SYBR Green Pro Taq HS Fluorescent Quantitative Detection Kit (AG11718). The cDNA loading amount of miRNA was 200 ng/well, and the primer concentration was 0.2 μM. The loading amount of cDNA for mRNA was 50 ng/well, and the primer concentration was adjusted appropriately within the range of 0.1-1.0 μM. The differential cycle number of cDNA was calculated by the △CT value to compare the gene expression among the samples of each group.

**Dot blot detection of m6A in RNA**

Saline sodium citrate (SSC) buffer (20×) and formaldehyde at a ratio of 3:1 were mixed and placed into a 1.5 mL EP tube to prepare 15× SSC buffer as the RNA stabilization buffer. The 0.45 μm PVDF membrane was immersed in methanol for 3 minutes, activated for 3 minutes, transferred to RNA-stabilized buffer and soaked for 15 minutes. Then, the membrane was dried in an oven at 55 °C for 10 minutes. Then, 1000 ng of total RNA and RNA stabilization buffer at a ratio of 1:2 (12 μL:24 μL) were mixed in a 1.5 mL EP tube, centrifuged at 1000 rpm for 1 min, heated in a metal bath at 65 °C for 15 min, and placed on ice. Spotting: A 20 μL pipette and a spotting tip were used to evenly spot the sample on the activated PVDF membrane. After sample application, the PVDF membrane was placed horizontally in an oven at 55 °C, dried for 1 h and washed 3 times with TBST solution on a shaker at room temperature. Then, the membrane was blocked in 5% BSA solution on a shaker at RT for 1 h and incubated with the prepared m6A primary antibody overnight at 4 °C. After washing, the membrane was probed with the corresponding secondary antibody for 1 hour at RT and detected with ECL reagents.

**Isolation and Culture of Primary Cardiac Fibroblasts (MCFs) and Drug Treatment**

After being sterilized by 75% ethanol, the suckling rat heart was removed and cut into tissue pieces with a uniform size of 1 m^2^ X 1 m^2^ X 1 m^2^ with ophthalmic scissors, and the residual blood was cleaned in cold PBS solution. Then, 0.08% trypsin (digestion solution) was added to digest the tissue fragments in a shaking water bath at 37 °C for 5 minutes. The digesting mixture was aspirated and filtered through a 70 μm cell sieve and transferred to a 15 mL BD tube. After that, complete medium containing 10% foetal bovine serum (FBS) was added to stop the digestion, and the cells were placed in a centrifuge at 1200 rpm for 6 minutes to obtain the digested cells. These steps were repeated until the tissue was digested completely. All collected cells were centrifuged at 1600 rpm for 8 min, resuspended in complete medium, and plated in a dish. After culture for 1.5 h, the adherent cells in the culture dish were recognized as primary cardiac fibroblasts and cultured in low-glucose medium. The cell incubator conditions were 37 °C, 5% CO2 and 95% humidity.

ISO was dissolved in PBS buffer solution, and the primary cardiac myofibroblasts were treated with the indicated concentration of ISO for 12 hours.

**EdU staining for cell proliferation analysis**

MCF proliferation was detected using the BeyoClick EdU-488 Cell Proliferation Detection Kit. The thymidine analogue EdU was added to MCFs and cultured for 2 hours. Subsequently, Alexa Fluor 488 was used to label the EdU incorporated in the process of cellular DNA replication by click reaction, and DAPI was used to stain the nucleus. The images were captured under a fluorescence microscope.

**Determination of cell viability**

Cell viability was determined by a 3-(4,5-dimethylthiazol-2-yl)-2,5-diphenyltetrazolium bromide (MTT) assay. MTT was prepared as a 5 mg/ml stock solution with PBS buffer. After drug treatment, the cells were treated with MTT solution at a final concentration of 5 μg/ml and placed in an incubator to continue culturing for 4 h. After removal of the medium, 150 μL of DMSO was added to each well. The plate was shaken for 10 minutes to completely dissolve the blue‒purple formazan crystals. Absorbance was measured at 490 nm.

**Cell transfection**

The cells were transfected with the transfection reagent LIPO and RNAFIT (Hanheng, Shanghai), and the dosage used in the 12-well plate was 2 μL/well. The transfection concentrations of siRNA, miRNA mimics and inhibitors were 50 nM, 50 nM and 100 nM, respectively. After the cells were transfected in low-glucose DMEM without serum and antibiotics for 12 hours, the complete medium was changed to continue culturing for 36 hours.

**Luciferase reporter assay**

The 3’UTR of Foxo4 including binding sites for miR-221-3p was amplified from mouse cDNA by PCR. A mutant 3’UTR fragment of Foxo4 which the mutations were in binding sites for miR-221-3p was also generated. The fragments including the 3’UTR regions (3’UTR-WT) or mutant 3’UTR regions (3’UTR-Mut) of Foxo4 were inserted into pSI-Check2 vector with a firefly luciferase reporter gene. Then the miR-221-3p mimics, NC mimics were transfected into 293T cells transfected with Foxo4 3’UTR-WT or Foxo4 3’UTR-Mut vectors respectively. After 48 h, cells were collected, and the firefly luciferase activities were determined using a Promega Dual-Luciferase system.

**Data processing and statistical methods**

Statistical analysis was performed by Student’s t test and one-way ANOVA using GraphPad Prism 8.0.
