# Supplementary material for: Heterogeneous nuclear ribonucleoprotein A2/B1 promotes myocardial fibrosis by regulating the miR‐221‐3p/FOXO4‐mediated inflammation
Source: Clin Transl Med. 2024 Mar 11;14(3):e1616. doi: 10.1002/ctm2.1616 (PMC10928344; doi:10.1002/ctm2.1616)
Supplement: Supplementary file 2 — Supporting Information [file CTM2-14-e1616-s001.docx]

**Table S1 The miRNAs that hnRNPA2B1 up-regulated and cardiac fibrosis related.**

| **HnRNPA2B1 up-regulated miRNA** | **Cardiac fibrosis-related miRNA** |
| --- | --- |
| miR-101 | let-7c-3p |
| miR-103a | miR-101-3p |
| miR-106b | miR-130a-3p |
| miR-107 | miR-133b |
| miR-10a | miR-145-3p |
| miR-1180 | miR-152-3p |
| miR-126 | miR-15b-5p |
| miR-1306 | miR-199b-5p |
| miR-1307 | miR-200c-3p |
| miR-130b | miR-210-3p |
| miR-140 | miR-223-3p |
| miR-151a | miR-29a-3p |
| miR-151b | miR-361-5p |
| miR-152 | miR-499a-5p |
| miR-17 | miR-891a-3p |
| miR-181a | miR-9-5p |
| miR-181c | miR-19a |
| miR-181d | miR-126 |
| miR-185 | miR-125b-5p |
| miR-186 | miR-210 |
| miR-18a | miR-21 |
| miR-18b | miR-23a-3p |
| miR-193b | miR-130a-3p |
| miR-194 | miR-34 |
| miR-20a | miR-29 |
| miR-210 | miR-197-5p |
| miR-221 | miR-1a-3p |
| miR-24 | miR-133a |
| miR-27b | miR-675 |
| miR-28 | miR-323a-3p |
| miR-301a | miR-202-3p |
| miR-30e | miR-217 |
| miR-320a | miR-133 |
| miR-320c | miR-384-5p |
| miR-324 | miR-200b |
| miR-331 | miR-367-3p |
| miR-345 | miR-181a |
| miR-3607 | miR-34b-3p |
| miR-3609 | miR-34c-5p |
| miR-361 | miR-34c-3p |
| miR-3651 | miR-433-3p |
| miR-378a | miR-214-5p |
| miR-381 | miR-21a-5p |
| miR-421 | miR-299a-5p |
| miR-423 | miR-154-5p |
| miR-425 | miR-134-5p |
| miR-4521 | miR-329-3p |
| miR-455 | miR-382-5p |
| miR-484 | miR-199a-5p |
| miR-497 | miR-431-5p |
| miR-500b | miR-146b-5p |
| miR-503 | miR-337-5p |
| miR-6516 | miR-409-3p |
| miR-660 | miR-411-3p |
| miR-6865 | miR-434-3p |
| miR-744 | miR-214-3p |
| miR-93 | miR-127-3p |
| miR-99b | miR-7033-5p |
| miR-125b | miR-541-5p |
| miR-149 | miR-199a-3p |
| miR-99a | miR-199b-3p |
|  | miR-338-5p |
|  | miR-99b |
|  | miR-126 |
|  | miR-221 |
